# The Sponge-Derived Brominated Compound Aeroplysinin-1 Impairs the Endothelial Inflammatory Response through Inhibition of the NF-κB Pathway

**DOI:** 10.3390/md20100605

**Published:** 2022-09-26

**Authors:** Isabel Vidal, Laura Castilla, Ana Dácil Marrero, Inés Bravo-Ruiz, Manuel Bernal, Inmaculada Manrique, Ana R. Quesada, Miguel Ángel Medina, Beatriz Martínez-Poveda

**Affiliations:** 1 Departamento de Biología Molecular y Bioquímica, Facultad de Ciencias, Universidad de Málaga, Andalucía Tech, 29071 Málaga, Spain; 2 Instituto de Investigación Biomédica de Málaga y Plataforma en Nanomedicina, IBIMA Plataforma BIONAND, 29590 Málaga, Spain; 3 CIBER de Enfermedades Raras (CIBERER), Instituto de Salud Carlos III, 28029 Madrid, Spain; 4 CIBER de Enfermedades Cardiovasculares (CIBERCV), Instituto de Salud Carlos III, 28029 Madrid, Spain

**Keywords:** aeroplysinin-1, marine-sponge-derived metabolites, endothelial cells, inflammation, NF-κB pathway, PI3K/Akt pathway, atherosclerosis

## Abstract

(+)-Aeroplysinin-1 (Apl-1) is a brominated compound isolated from the marine sponge *Aplysina aerophoba* that exhibits pleiotropic bioactive effects, impairing cell growth in cancer cells, inhibiting angiogenesis in vitro and in vivo and modulating the redox status of different cell types, among other reported activities. In addition to the aforementioned effects, the anti-inflammatory potential of this natural compound was explored in previous work of our laboratory, but the mechanism of action underlying this effect was not described. In this work, we delve into the anti-inflammatory effect of Apl-1 in the context of vascular endothelial cells in vitro, providing new data regarding the molecular mechanism underlying this activity. The characterization of the mechanism of action points to an inhibitory effect of Apl-1 on the NF-κB pathway, one of the main axes involved in endothelial response during inflammatory events. Our results show that Apl-1 can inhibit the expression of pro-inflammatory genes in tumor necrosis factor alpha (TNF-α)- and lipopolysaccharide (LPS)-stimulated human umbilical vein endothelial cells (HUVECs), targeting the nuclear factor kappa B subunit (NF-κB) pathway through a mechanism of action involving the inhibition of I kappa B kinase (IKK) complex phosphorylation and RelA/p65 nuclear import. In addition, Apl-1 prevented the phosphorylation of Akt induced by TNF-α in HUVECs, probably supporting the inhibitory effect of this compound in the NF-κB pathway. Experimental evidence reported in this work opens the door to the potential pharmacological use of this compound as an anti-inflammatory agent in diseases that course with a pathological endothelial response to inflammation, such as atherosclerosis.

## 1. Introduction

The endothelium represents the first barrier in blood vessels for migration of immune cells to inflammation sites. Therefore, the response of endothelial cells (ECs) to inflammatory signals, manifested in phenotypic changes involving the expression of pro-inflammatory cytokines, chemotactic factors and adhesion molecules, is critical for the promotion of the inflammatory process, allowing the recruitment of blood immune cells into the arterial intima [1]. Evidence shows that a deregulated inflammation in the endothelium is linked to endothelial dysfunction, being associated to the development of several pathological conditions, such as atherosclerosis, aortic aneurysm or rheumatoid arthritis [1,2,3,4,5,6].

Dynamic phenotypic changes in ECs are modulated by intracellular signaling cascades that respond to extracellular stimuli, with the NF-κB pathway being one of the central axes in the activation of the inflammation-driven phenotype. It is well accepted that the NF-κB pathway is activated in response to a large diversity of soluble and membrane-bound extracellular ligands that signal through their respective receptors. They are mainly members of the tumor necrosis factor receptor (TNFR), Toll-like receptor (TLR) and interleukin-1 receptor (IL-1R) superfamilies and are responsible for activating the transcription of a huge number of target genes that are involved in a great variety of biological processes, including immunity, stress responses, inhibition of apoptosis and inflammation [7,8]. In addition, two major activation pathways, the canonical (triggered by TNF-α, IL-1 or LPS) and the alternative (triggered by CD40 ligand, CD40L) pathways, have been described for NF-κB, and the relevance of these pathways depends on the cell type and context [9]. Although both the alternative pathway and a shear-stress-dependent mechanism have been related to the NF-κB activation in ECs, the strongest response for these cells in vitro has been that described for TNF-α, developed through the canonical pathway and triggering the expression of target genes [9,10].

During the initiation and progress of atherosclerotic lesions, the inflammatory events activated in ECs promote the expression of cell adhesion molecules and pro-inflammatory soluble factors, leading to the recruitment of immune cells (including monocytes/macrophages, neutrophils and lymphocytes) which invade the intima layer, promoting and sustaining inflammation in the vessel wall [1,11,12]. Although lipid-lowering therapies, such as hydroxy-3-methylglutaryl coenzyme A (HMG-CoA) reductase inhibitors (statins), have been extensively used in atherosclerosis treatment, recent findings evidenced that the modulation of the pro-inflammatory context in atheroma lesions represents a powerful therapeutic strategy that could be complementary to the use of classical lipid-targeting approaches against atherosclerosis [13].

In this context, the pharmacological potential of natural products to target atherosclerosis emerges, supported by their pleiotropic bioactivity as anti-inflammatory, antioxidant, lipid-lowering and anti-thrombotic molecules [14]. Of note, pharmacological interest in marine organisms as a rich source of molecules for the research and development of potential anti-atherosclerotic drugs has gained attention in recent years [15]. Among them, the huge diversity of secondary metabolites derived from marine sponges represents a unique resource of chemical structures and bioactivities [16].

**Figure 1:**
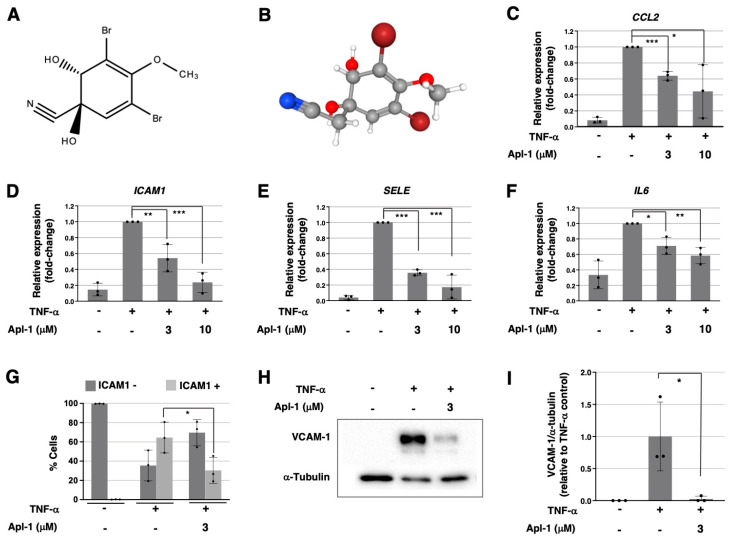
(**A**) Molecular structure and (**B**) three-dimensional structure of (+)-Apl-1 (downloaded from PubChem); (**C**–**F**) qPCR-measured relative expression of (**C**) *CCL2*, (**D**) *ICAM1*, (**E**) *SELE* and (**F**) *IL6* genes in HUVECs under indicated treatments for 6 h; in each case, expression was relativized to the actin beta gene (*ACTB*) and normalized to the TNF-α positive control; (**G**) Flow-cytometry-measured ICAM-1 levels in HUVECs under indicated treatments for 16 h; percentage of expressing and non-expressing ICAM-1-cells (ICAM+ and ICAM1-) are represented; (**H**) Representative Western blot images of vascular adhesion molecule 1 (VCAM-1) and α-tubulin levels in HUVECs under indicated treatments for 16 h; (**F**) Densitometric quantification of VCAM-1 and α-tubulin bands in (**G**) and two additional independent experiments, VCAM-1 signals were relativized to α-tubulin and normalized to the TNF-α positive control. In panels (**C**–**G**,**I**), means and standard deviations of three independent replicates are represented and statistical analyses were performed through *t*-tests; * *p* < 0.05, ** *p* < 0.01, *** *p* < 0.001.

The bromo-tyrosine alkaloid (+)-aeroplysinin-1 (Apl-1) (Figure 1A, B) is a secondary metabolite biosynthesized by marine sponges including *Aplysina aerophoba* and other species from the order Verongida distributed in tropical to temperate seawaters along the Mediterranean Sea and the Atlantic and Pacific Oceans [17]. The bioactive properties of Apl-1 were previously reported by us and other groups, evidencing the pleiotropic role of this compound in different pathological contexts (reviewed in [17,18]). Interestingly, Apl-1 was described as a potent anti-angiogenic compound in vitro and in vivo, inhibiting essential steps of the angiogenic process, such as EC proliferation, migration, extracellular matrix invasion and tubular-like reorganization [19]. Thus, the anti-angiogenic capability of Apl-1, together with its reported anti-tumoral activity in vitro [20,21,22], point to the therapeutic potential of this natural compound in a context of angiogenesis-dependent tumors.

However, in addition to the anti-angiogenic and anti-tumoral activities of Apl-1, the potential role of this natural compound as an anti-inflammatory molecule was reported in a previous study of our laboratory, where the capability of Apl-1 to impair the expression of inflammation-related targets in ECs and monocytes was described, suggesting a putative pharmacological application of this molecule to inflammation-dependent diseases [23]. Despite this proposed role of Apl-1 as an anti-inflammatory compound, its mechanism of action and the signaling pathways underlying this effect in the endothelium have not been described yet. 

In this work, we explore the anti-inflammatory potential of Apl-1 in a context of endothelial inflammation, identifying for the first time the impairment of the NF-κB pathway as a crucial molecular target in the mechanism of action underlying the anti-inflammatory activity of Apl-1 in human ECs.

## 2. Results

### 2.1. Aeroplysinin-1 Inhibits the Expression of Inflammation-Related Molecules in ECs

In response to a pro-inflammatory signal, endothelial cells activate the expression of inflammatory molecules implicated in immune cells’ chemoattraction and adhesion, phenotypic changes related to the early development of vascular pathologies such as atherosclerosis. To determine the effect of Apl-1 in a context of inflammation-related endothelial activation, HUVECs were induced with TNF-α in the presence of different doses of Apl-1 and the expression of genes encoding inflammatory molecules was measured. As seen in Figure 1C, the induction of HUVECs with TNF-α strongly activated the expression of C-C motif chemokine ligand 2 (*CCL2* gene), encoding monocyte chemoattractant protein-1 (MCP1), and this expression was significantly reduced in the presence of Apl-1 in a dose-response manner (Figure 1C). In the same way, Apl-1 treatments in HUVECs resulted in the reduction of the TNF-α-induced expression of the intercellular adhesion molecule 1 (*ICAM1*), selectin E (*SELE*) and interleukin 6 (*IL6*) genes, encoding ICAM-1, E-selectin and IL-6, respectively (Figure 1D–F). 

In addition, changes observed at the mRNA level for the adhesion-molecule-encoding genes were reflected at the protein level in HUVECs. As seen in Figure 1G, Apl-1 inhibited the TNF-α-induced exposition of ICAM-1 in the cellular membrane of ECs (Figure 1G). In addition, the presence of VCAM-1 in HUVECs was activated by TNF-α, but Apl-1 treatment strongly abrogated its expression (Figure 1H,I).

**Figure 2:**
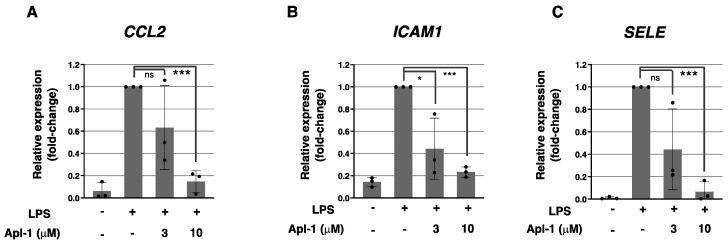
(**A**–**C**) qPCR-measured relative expression of (**A**) *CCL2*, (**B**) *ICAM1* and (**C**) *SELE* genes in HUVECs under indicated treatments for 6 h; in each case, expression was relativized to the *ACTB* gene and normalized to the LPS positive control; means and standard deviations of three independent replicates are represented and statistical analyses were performed through *t*-tests; * *p* < 0.05, *** *p* < 0.001.

The inhibitory effect of Apl-1 on the expression of inflammation-related molecules in the EC did not seem to be specific to the TNF-α induction, since similar results were obtained when the response of HUVECs to LPS, another pro-inflammatory signal in the endothelium, was measured. As shown in Figure 2, Apl-1 significantly inhibited the LPS-induced expression of *CCL2*, *ICAM1* and *SELE* in HUVECs, suggesting that this compound could be targeting a common element in the activation pathways of both the TNF-α and LPS pro-inflammatory signals.

### 2.2. Apl-1 Impairs NF-κB Nuclear Translocation in Response to TNF-α in ECs

**Figure 3:**
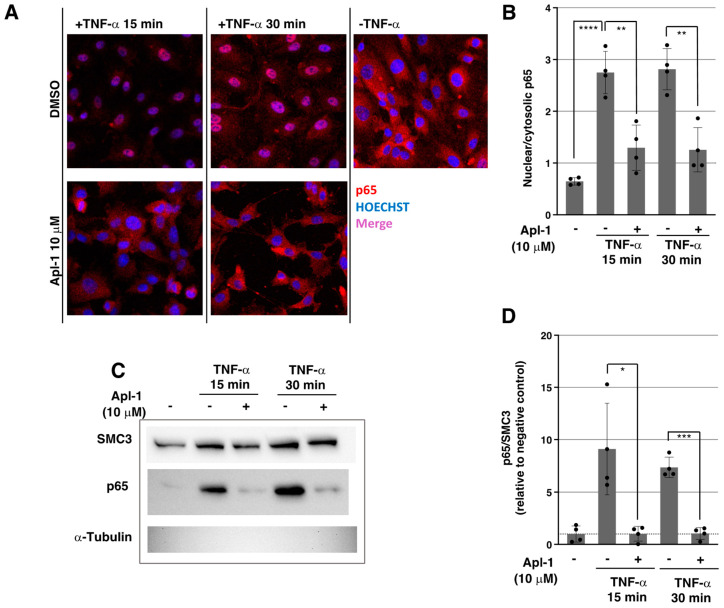
(**A**) Representative confocal images of RelA/p65 (red) merged with Hoechst (blue) in HUVECs under indicated treatments (20× magnification); (**B**) The relative nuclear/cytosolic RelA/p65 signal was measured in confocal images of four independent experiments and means and standard deviations are represented; (**C**) Representative Western blot images of RelA/p65, structural maintenance of chromosome 3 (SMC3, nuclear protein control) and α-tubulin (cytosolic protein control) levels in nuclear fractions of HUVECs under indicated treatments; (**D**) Densitometric quantification of (**C**) and three additional independent experiments, RelA/p65 bands were relativized to SMC3 bands and normalized to the negative control (unstimulated cells) and means and standard deviations of four independent replicates are represented; statistical analyses were performed through *t*-tests; * *p* < 0.05, ** *p* < 0.01, *** *p* < 0.001, **** *p* < 0.0001.

The NF-κB pathway is one of the main intracellular axes implicated in the activation of the inflammatory response in ECs and other cell types [24,25]. Multiple extracellular pro-inflammatory signals converge in the activation of this pathway, promoting the transcription of a high number of target genes, including *CCL2*, *ICAM1*, *VCAM1*, *SELE* and *IL6*. According to the observed inhibition in the TNF-α- and LPS-induced expression of these inflammatory molecules by Apl-1 in HUVECs, we next studied the effect of this compound in NF-κB nuclear translocation, one of the crucial steps in the activation of the pathway, to analyze if the signaling was impaired. As shown in Figure 3A, TNF-α rapidly induced the nuclear translocation of cytosolic RelA/p65, one of the proteins that conforms the NF-κB dimer in the canonical pathway [9]. However, the localization of RelA/p65 in Apl-1-treated HUVECs stimulated with TNF-α for 15 and 30 min was mainly restricted to the cytosolic compartment, similar to ECs in absence of TNF-α induction, which indicates that this compound prevented the nuclear localization of RelA/p65 in HUVECs at the studied timepoints (Figure 3A). Quantification of the immunofluorescence images showed a significant reduction in the ratio of nuclear/cytosolic RelA/p65 signals in Apl-1-treated HUVECs (Figure 3B), pointing to the involvement of this molecule in the inhibition of the NF-κB pathway.

In addition, the same inhibitory effect was observed in Western blots of nuclear fractions of TNF-α-induced HUVECs, revealing once again that Apl-1 strongly inhibited the translocation of RelA/p65 to the nucleus (Figure 3C,D), a critical step implicated in NF-κB pathway activation.

### 2.3. Apl-1 Inhibits IKKα/β Phosphorylation in ECs

**Figure 4:**
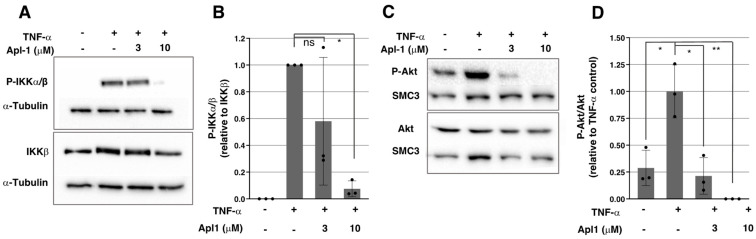
(**A**) Representative Western blot images of phosphorylated IKKα/β (P-IKKα/β), total IKKβ and α-tubulin levels in HUVECs under indicated treatments; (**B**) Densitometric quantification of (**A**) and two additional independent experiments, P-IKKα/β bands were relativized to total IKKβ bands and normalized to TNF-α positive control; (**C**) Representative Western blot images of phosphorylated Akt (P-Akt), total Akt and SMC3 levels in HUVECs under indicated treatments; (**D**) Densitometric quantification of (**C**) and two additional independent experiments, P-Akt bands were relativized to total Akt bands and normalized to TNF-α positive control; in (**B**,**D**), means and standard deviations of three independent replicates are represented; statistical analyses were performed through *t*-tests; * *p* < 0.05, ** *p* < 0.01.

Nuclear translocation of NF-κB in response to activating signals is regulated by upstream intracellular elements in the pathway. To better define the inhibitory role of Apl-1 in the NF-κB nuclear import in endothelium, the phosphorylation of the IKKα/β complex was studied in order to determine its activation state. IKKα and IKKβ conform a protein complex that, together with NF-κB essential modulator (NEMO), transduces the activation signal from TNFR to NF-κB in the canonical pathway, mediating the degradation of the cytosolic NF-κB inhibitor alpha (IκBα), a protein responsible for NF-κB-cytosolic retention [9]. As shown in Figure 4A,B, in HUVECs induced with TNF-α, IKKα/β was rapidly phosphorylated, but Apl-1 treatment significantly reduced its phosphorylation, pointing to the inhibition of upstream intracellular elements in the NF-κB pathway in the presence of this compound.

### 2.4. Activation of the PI3K/Akt Pathway Is Inhibited by Apl-1 in ECs

The phosphatidylinositol 3-kinase (PI3K)/Akt pathway is involved in the regulation of numerous cellular processes [26], and a crosstalk of this cascade with the NF-κB pathway has been described in different cell types, promoting direct phosphorylation of the IKK complex and consequently eliciting NF-κB nuclear translocation and transcriptional activity [27,28]. Figure 4C,D show that TNF-α induced Akt phosphorylation in HUVECs, suggesting that the PI3K/Akt pathway could be involved in the NF-κB-mediated inflammatory response in these cells by a crosstalk of both pathways. According to the previous results of our laboratory, Apl-1 inhibits Akt phosphorylation in response to serum stimulation in ECs [23], but the effect of this compound on the TNF-α-induced Akt activation is not described. As seen in Figure 4C,D, Apl-1 strongly reduced Akt phosphorylation in response to TNF-α, suggesting that the inhibitory activity of Apl-1 in the PI3K/Akt pathway could contribute to the observed impairment of the NF-κB pathway in ECs.

## 3. Discussion

In a previous work of our research group, the anti-inflammatory potential of Apl-1 in ECs and monocytes was reported [23]. However, the mechanism of action underlying this bioactivity was not then unveiled. In this study, we describe for the first time that Apl-1 impairs the response of ECs to some classical pro-inflammatory signals, such as TNF-α and LPS, through the inhibition of the NF-κB pathway by a mechanism of action involving prevention of IKK complex phosphorylation and concomitant RelA/p65 nuclear translocation.

The inhibitory effect of Apl-1 in the expression of inflammatory molecules in ECs was firstly described by us [23]. In that work, Apl-1 was shown to reduce the expression of MCP1, thrombospondin 1 (TSP1), cyclooxygenase 2 (COX-2) and interleukin 1 alpha (IL-1α), and different experimental conditions were used to study these effects. For instance, MCP1 and TSP1 expressions were studied in non-stimulated ECs, whereas COX-2 expression was elicited by tetradecanoyl phorbol acetate (TPA) (a protein kinase C, PKC, activator), and IL-1α expression was induced with the pro-inflammatory peptide SLIGKV (a protease-activated receptor-2, PAR2, agonist). In the present work, we tried to reproduce a more physiological context for the study of the endothelial inflammatory response in vitro, using TNF-α as a pro-inflammatory signal in the different experiments. According to previous results, Apl-1 clearly impairs the activation of the inflammatory response in ECs, in this case upon TNF-α stimulation, reducing the expression of different cell adhesion molecules (ICAM-1, VCAM-1 and E-selectin) and soluble factors (MCP1, IL-6). Interestingly, the inhibitory effects of Apl-1 in the expression of genes in response to TNF-α were observed with 6 h of treatment at 3 μM of the compound, a dose lower than the IC_50_ reported for Apl-1 in HUVECs (4.7 μM at 3 days of treatment) [23]. The role of ICAM-1, VCAM-1 and E-selectin in ECs is related to the recruitment of blood immune cells, such as lymphocytes and monocytes, to the vessel wall, through direct binding of these cells to endothelium and allowing diapedesis. In addition, MCP1 is one of the main chemokines mediating leukocytes’ chemoattraction to inflammation sites. Thus, our data strongly suggest that Apl-1 could impair the recruitment and binding of monocytes and T lymphocytes to the endothelium, thus preventing inflammation in the vascular wall. Other critical events occurring in chronic inflammatory diseases are the increased permeability of the endothelium and vascular leakage [29]. IL-6 is a pro-inflammatory cytokine with pleiotropic functions, which promotes the endothelial barrier breakdown and vascular leakage [30]. Therefore, the observed reduction of IL-6 expression in ECs by Apl-1 could suggest a putative involvement of this compound in the prevention of vascular leakage. Further studies are needed to validate the role of Apl-1 in immune cell recruitment and vascular permeability.

Of note, the reduced expression of pro-inflammatory molecules in the presence of Apl-1 is observed under different stimuli (TNF-α, LPS or SLIGKV peptide, this last treatment described in [23]) that signal through different extracellular receptors, supporting the hypothesis that Apl-1 could be targeting a common element of these activation pathways, rather than directly inhibiting at the ligand/receptor level. In this work, we show that Apl-1 clearly impairs NF-κB pathway activation, inhibiting the cytosol-to-nucleus shift of RelA/p65 in ECs. Nuclear localization of RelA/p65 (one of the subunits of the NF-κB dimer in the canonical pathway) is critical for the transcriptional activation of the target genes, and its translocation from the cytosol occurs very fast when the pathway is activated by extracellular stimuli. Our data suggest that Apl-1 modulates the pathway in a direct manner, since the effect of this compound in the reduction of the nuclear/cytosolic ratio of RelA/p65 is observed after a short-time treatment with Apl-1 in ECs (1 h pre-treatment and 15–30 min of TNF-α stimulation). Consistent with this, we report in this work that the phosphorylation of IκB kinases α and β (IKKα/β), an essential step leading to NF-κB nuclear import, is reduced in ECs in presence of Apl-1, and this inhibition is also observed after a short-time incubation with the compound (1 h). IKKα/β are the catalytic subunits of IKK complex which promote the release of NF-κB dimer from the cytosolic protein IκBα and its nuclear transport, secondary to direct phosphorylation of IκBα and subsequent ubiquitination and degradation by the proteasome. Therefore, the inhibition of IKKα/β phosphorylation by Apl-1 would result in the impairment of the NF-κB pathway in ECs. In the pathogenesis of atherosclerosis, dysfunctional vascular endothelium is considered as a driver element. Indeed, a chronic inflammatory response in the endothelium is a critical early event of the disease, contributing as well to sustaining the atherosclerotic plaque progression [1,31]. Of note, a critical role of the endothelial NF-κB pathway was evidenced in animal models of atherosclerosis, where genetic abrogation of the signaling prevented the development of atheroma lesions in Western-diet-fed apolipoprotein E knock-out (*ApoE*^−/−^) mice [5]. The discovery of the inhibitory effect of Apl-1 in NF-κB signaling in ECs reveals a putative therapeutic role of this natural compound in atherosclerosis, although further in vivo experiments are needed to validate this hypothesis.

**Figure 5:**
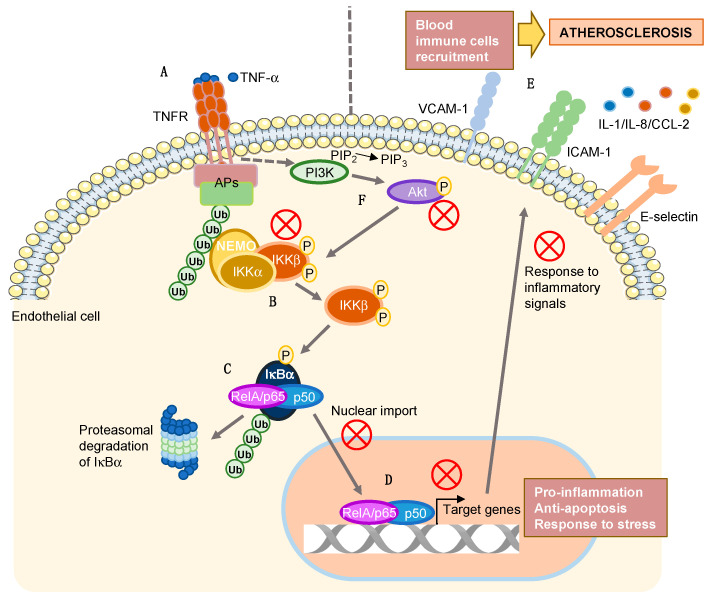
Summary of the different inhibitory effects of Apl-1 in the endothelial response to TNF-α revealed in the present study. Red blades represent steps targeted by Apl-1. The canonical NF-κB pathway is simplified in the scheme. (**A**) Pro-inflammatory signal (TNF-α) activates its receptor in EC; (**B**) IKK complex is activated downstream TNFR; (**C**) Phosphorylated IKK promotes degradation of IκBα, releasing the NF-κB dimer RelA/p65-p50, and allowing its nuclear translocation and transcriptional activity (**D**); (**E**) Adhesion molecules are exposed in the surface of EC, and cytokines and chemokines are released, promoting the inflammatory response in the cell; (**F**) PI3K/Akt pathway is activated in response to TNF-α, promoting the phosphorylation of IKK complex. APs, adaptor proteins; Ub, ubiquitin residue; P, phosphorylated residue; TNF-α, tumor necrosis factor-alpha; TNFR, TNF-α receptor; NEMO, NF-κB essential modulator; IKK, I kappa B kinase; IκBα, NF-κB inhibitor alpha; PI3K, phosphoinositide 3-kinase; VCAM-1, vascular adhesion molecule-1; ICAM-1, intercellular adhesion molecule-1; IL, interleukin. The figure was partly generated using Servier Medical Art, provided by Servier, licensed under a Creative Commons Attribution 3.0 unported license.

In addition to the canonical activation of NF-κB activity, it was described that several signaling pathways can establish a crosstalk with the NF-κB pathway, enhancing its activation and modulating the response of ECs to inflammation in different pathological contexts [24,32,33]. This is the case of the PI3K/Akt pathway, which was reported to affect the NF-κB pathway in different checkpoints depending on cell type. Hence, Akt was shown to indirectly induce NF-κB nuclear translocation by direct phosphorylation of the IKK complex in response to different signals, such as TNF and platelet-derived growth factor (PDGF) [27,28]. In addition, Akt promotes the transcriptional function of NF-κB by stimulating RelA/p65 transactivation through an IκB-independent mechanism involving IKK and p38 [34]. In ECs, we observed that the PI3K/Akt pathway is rapidly activated in response to TNF-α, suggesting a possible contribution of this pathway to the TNF-α-induced NF-κB activation in these cells. However, Apl-1 clearly abrogates Akt phosphorylation in response to TNF-α, indicating the interference of this compound in the pathway. In previous studies of our group, we described that Apl-1 inhibits PI3K/Akt pathway activation in ECs stimulated with serum [35], and the interference of the compound in this pathway was recently described to be involved in the antitumoral activity of Apl-1 in prostate cancer and leukemia cells in vitro [20]. Thus, we postulate that the reduction of TNF-α-induced Akt phosphorylation by Apl-1 in ECs is involved in the mechanism of action of Apl-1 to inhibit the endothelial response to inflammation, since the impairment of this pathway could downregulate both NF-κB nuclear translocation and transcriptional activity through reduced IKK phosphorylation. Although we cannot discard that Apl-1 could be affecting additional targets in ECs, our results suggest that the inhibition of the PI3K/Akt pathway could be a crucial point in the mechanism of action underlying the Apl-1-mediated impairment of the endothelial response to inflammatory stimuli (Figure 5). Interestingly, it was suggested that targeting the NF-κB pathway through inhibition of interplaying signaling cascades should constitute an effective and safe therapeutic strategy for inflammation-associated diseases, since damping of NF-κB, rather than a complete blockage of the activating receptors, would allow the modulation of physiological host defense and repair mechanisms [24].

Interestingly, the anti-inflammatory properties of other natural brominated compounds have been described in the literature. Ahmad and collaborators [36] showed that brominated indoles from a marine mollusk can impair NF-κB nuclear translocation in RAW 264.7 macrophages stimulated with LPS, suggesting that the presence of the bromide atom and its position in the molecule is critical for the anti-inflammatory effect in these cells [36]. In addition, McCauley and collaborators [37] performed a pharmacological screening with extract fractions from 80 species of Indonesian marine sponges, focused on the search for molecules targeting the bacterial type III secretion system (T3SS). In that work, they identified a fraction based in its ability to inhibit T3SS-driven NF-κB activation in HEK293 cells in a panel of human cell lines. Of note, in that fraction, seven bromo- and iodo-tyrosine-derived alkaloids were obtained, enisorines A and C-E and two hemibastadinol analogues, all of them containing the moloka’iamine core substructure [37]. Although further structure–function studies would be needed to get a conclusion, based on data available in the scientific literature, it is worthy to suggest that the herein reported inhibition of the NF-κB pathway by Apl-1, another bromo-tyrosine-derived compound, could be related to the presence of this type of bromide-containing structure. Future studies on structure–activity relationships will be required to further assess this hypothesis.

In addition to the direct role of the endothelium in initiation and sustaining of atherosclerosis, the inflammatory context in atheroma lesions elicits the activation of angiogenesis from *vasa vasorum*. Hence, microvessel expansion within the arterial wall was proposed as an important feature in the initiation, progression and rupture of the plaque [38,39]. In fact, the inhibition of plaque neovascularization was shown to reduce atherosclerosis, promoting a beneficial effect in plaque stability [40,41]. Therefore, targeting the response of the endothelium to both pro-inflammatory and pro-angiogenic signals was suggested as a powerful strategy in the future therapy of atherosclerosis [39]. Interestingly, besides the herein described anti-inflammatory potential of Apl-1 through inhibition of NF-κB signaling in endothelial cells, the anti-angiogenic activity of this natural compound was reported in vivo and in vitro [19], pointing to a dual role of Apl-1 in ECs, impairing the inflammatory response and angiogenesis. This observation reinforces the suggested pharmacological role of Apl-1 as a candidate for the treatment of atherosclerosis, motivated by the pleiotropic bioactivity exhibited by this molecule. Further in vivo experiments are needed to validate this hypothesis.

Our research group has been interested in the study of the bioactivity of Apl-1 in endothelial cell function since 2001, when we first described the anti-angiogenic activity of this brominated compound in vitro and in vivo [19]. The mechanism of action of its anti-angiogenic effect was further described in different works, where we showed that Apl-1 induces apoptosis in proliferating ECs by activating the intrinsic (mitochondrial) pathway [42], by a mechanism involving PI3K/Akt and extracellular-signal-regulated protein kinase (ERK)1/2 pathways inhibition [35]. More recently, the capability of Apl-1 to modulate the redox balance in ECs was reported by us, shedding new light on the previously described anti-angiogenic effect of this compound [43]. In addition to its antiangiogenic potential, the putative anti-inflammatory role of Apl-1 in the endothelium and monocytes was evidenced by our research group [23], although the exact mechanism of action of this effect was not described. Now, we demonstrate for the first time that Apl-1 inhibits the NF-κB pathway in ECs, downregulating the transcriptional response of these cells to TNF-α and LPS stimuli in vitro by a direct mechanism of action that involves impairment of IKK phosphorylation (and subsequent NF-κB nuclear import) and inhibition of the PI3K/Akt pathway. Although the IC_50_ value of Apl-1 in HUVECs reported in [23] was 4.7 μM, it is worthy to highlight that the inhibitory effects of Apl-1 in the endothelial response to inflammation were observed in most of the assays at 3 μM, a dose lower than its IC_50_, and the incubation times of the shown experiments were much lower than those used for the determination of the IC_50_ (3 days) [23]. Figure 5 summarizes the different inhibitory effects of Apl-1 on responses to inflammatory signals revealed in the present study. It is noteworthy that the inhibition of the PI3K/Akt pathway seems to be a recurrent event in the bioactivity of Apl-1, pointing to this axis as a critical element in the molecular mechanism of action of this pleiotropic natural compound in ECs.

## 4. Materials and Methods

### 4.1. Cell Culture and Treatments

Human umbilical vein endothelial cells (HUVECs) were purchased from Lonza (Basel, Switzerland) and maintained in EGM-2 medium (Lonza) in a 5% CO_2_ humid atmosphere at 37 °C; cells were used until pass 9, following manufacturer recommendations. Apl-1 was purchased from Abcam (Cambridge, UK) and solubilized in dimethyl sulfoxide (DMSO). All treatments were performed in HUVECs at 80% confluence, in EGM-2 medium, at doses and times indicated in each assay; an equivalent DMSO (vehicle) concentration was added in control conditions.

### 4.2. Gene Expression Analysis by Real-Time Quantitative PCR

HUVECs were treated with different concentrations of Apl-1 or vehicle (DMSO) for 1 h before induction with 20 ng/mL TNF-α or 1 μg/mL bacterial lipopolisaccharides (LPS). After 6 h, cells were harvested and RNA was extracted using Tri Reagent (Merk-Sigma-Aldrich, Darmstadt, Germany) and a Direct-zol RNA MiniPrep kit (Zymo Research, Irvine, CA, USA). After quantification in a NanoDrop One spectrophotometer (Thermo Fisher Scientific, Waltham, MA, USA), mRNA was reverse-transcribed to cDNA using PrimeScript™ RT Master Mix (Takara Bio INC., Kusatsu, Japan). Real-time quantitative PCR (rt-qPCR) was performed using TB Green Premix Ex Taq II (Takara Bio INC.) and predesigned custom primers (KiCqStart, Merk/Sigma-Aldrich) in an Eco Illumina device (Illumina; San Diego, CA, USA). In each case, mRNA expression was relativized to *ACTB* gene (β-actin) expression, and values were normalized with respect to expression levels in the positive control condition (cells induced with TNF-α in the presence of DMSO) in order to calculate fold change. Three independent replicates of these assays were performed, with technical duplicates in each assay.

### 4.3. Protein Detection by Western Blot

Treatments in HUVECs were performed under different conditions for protein detection by Western blot. For VCAM-1 detection in whole cell lysates, DMSO or Apl-1 at indicated concentration were added to cells during overnight incubation (16 h) in the presence or absence of TNF-α; for detection of RelA/p65 nuclear translocation by Western blot, HUVECs were treated with DMSO or Apl-1 during 1 h prior to TNF-α induction for indicated times and nuclear extracts were obtained using a NE-PER Kit (Thermo Fisher Scientific) following manufacturer’s indications; for total IKK/phospho-IKK and Akt/phospho-Akt detection in whole cell lysates, HUVEC were incubated in the presence of DMSO or Apl-1 for 1 h and then induced with TNF-α for additional 15 min. Whole-cell lysates were performed in lysis buffer (0.5 M Tris-HCl, pH 6.8; 12% SDS; 10% glycerol). In nuclear and whole-cell lysates, protein concentration was quantified using DC Protein Assay (Bio-Rad Laboratories; Hercules, CA, USA), and β-mercaptoethanol and bromophenol blue were added at final concentrations of 5% and 0.2% in samples, respectively. After denaturalization at 95 °C for 5 min, 30 μg of total protein was subjected to SDS-PAGE electrophoresis and transferred to nitrocellulose membranes. After blocking in TBS-T (20 mM Tris pH 7.6, 137 mM NaCl, 0.1% Tween-20) containing 10% non-fatty dry milk, membranes were hybridized with primary antibodies overnight at 4 °C. Phospho-Akt (Ser473) rabbit mAb (#9271), Akt (#9272), SMC3 (D47B5) rabbit mAb (#5696), phospho-IKKα/β (Ser176/180; 16A6) rabbit mAb (#2697), IKKβ (D30C6) rabbit mAb (#8943) and α-Tubulin (DM1A) mouse mAb (#3873) antibodies were provided by Cell Signaling Technology (Danvers, MA, USA). VCAM-1 mouse mAb (sc-13160) and RelA/p65 mouse mAb (sc-8008) were purchased from Santa Cruz Biotechnology (Dallas, TX, USA). Membranes were washed in TBS-T and incubated for 1 h with secondary horseradish peroxidase-conjugated secondary antibodies at room temperature (donkey anti-rabbit IgG HRP-linked was from Merck, and horse anti-mouse IgG HRP-linked was from Cell Signaling Technology). Immunoreactive bands were detected with SuperSignal West Pico Chemiluminescent Substrate (Pierce; Rockford, IL, USA) in a Chemidoc XRS System (Bio-Rad Laboratories; Hercules, CA, USA). Densitometric quantification of the bands was performed with Fiji software [44] and values are expressed as the ratio of phosphorylated/non-phosphorylated protein relative to the positive control of induction (DMSO plus TNF-α). Nuclear RelA/p65 is expressed as the ratio of p65/SMC3 signals in nuclear extracts. VCAM-1 is expressed as the ratio of VCAM-1/α-tubulin. At least three independent replicates were performed of these assays.

### 4.4. ICAM-1 Detection by Flow Cytometry

HUVECs were treated with Apl-1 or DMSO for 1 h prior to TNF-α induction. After 16 h in the presence of treatments and TNF-α, cells were washed in PBS (137 mM NaCl, 2.7 mM KCl, 10 mM Na_2_HPO_4_, 1.8 mM KH_2_PO_4_, pH 7.4), harvested by trypsinization and transferred to an Eppendorf tube. After that, cells were washed twice with cold PBS supplemented with 10% fetal bovine serum (FBS). Cells were transferred to 96-well plate and incubated with 200 μL of the primary antibody solution (10 μg of anti-ICAM-1 in PBS + 10% FBS) for 1 h at 4 °C. Cells were washed twice with cold PBS + 10% FBS and then incubated with 200 μL of secondary antibody solution (10 μg of anti-mouse Alexa Fluor 568 in PBS + 10% FBS) for 30 min at 4 °C. After this incubation, cells were washed twice with cold PBS + 10% FBS and analyzed by flow cytometry (BD FACSVerse™). Three independent experiments were carried out for the indicated conditions.

### 4.5. RelA/p65 Immunofluorescence in HUVECs

For detection of RelA/p65 nuclear translocation by immunofluorescence, HUVECs were grown onto gelatin-coated coverslides in a culture dish. Then, cells were incubated with Apl-1 or DMSO for 1 h, and induced with TNF-α for an additional 15 or 30 min. Cells were fixed in formalin solution (Merck-Sigma-Aldrich) and RelA/p65 was detected by immunofluorescence. Briefly, fixed cells were permeabilized in Triton X-100 0.5% in PBS, and then blocked in 5% BSA in PBS. Cells were then incubated for 1 h at room temperature with RelA/p65 mouse mAb (sc-8008; Santa Cruz Biotechnology), washed in PBS, and then incubated with Alexa Fluor 568-linked anti-mouse IgG antibody (A11004, Invitrogen, Thermo Fisher Scientific, Waltham, MA, USA). In the last washing step, 1 μg/mL Hoechst 33342 was added and coverslides were mounted in glass slides using 1:10 (vol-vol) glycerol–water solution. Preparations were observed under fluorescence–confocal microscopy in a Leica TCS SP8 MP (Leica Microsystems, Wetzlar, Germany) using Leica Application Suite X software (Leica Microsystems) and images were obtained at 20× magnification. Quantification of nuclear/cytosolic p65 signal was performed with Fiji software [44] in at least 5 images of each condition. Three independent replicates were performed of this assay.

### 4.6. Statistical Analysis

Replicates from at least three independent experiments were performed in all assays. Statistical analyses of obtained results were performed through *t*-tests with GraphPad Prism software and *p* values < 0.05 were considered statistically significant. *p* values were represented as * *p* < 0.05, ** *p* < 0.01, *** *p* < 0.001.

## Data Availability

Not applicable.
